# Comparative benefit from small tumour size and adjuvant chemotherapy: clues for explaining breast cancer mortality decline

**DOI:** 10.1186/1471-2407-14-702

**Published:** 2014-09-24

**Authors:** Romano Demicheli, Federico Ambrogi

**Affiliations:** Scientific Directorate, Fondazione IRCCS Istituto Nazionale Tumori, Via Venezian 1, 20133 Milano, Italy; Department of Clinical Sciences and Community Health, Università degli Studi di Milano, Milano, Italy

## Abstract

**Background:**

Breast cancer mortality steadily declined from the 1990s and this has been attributed to early detection and/or to improvements in therapy. Which of those two has had the greater impact is a subject of contention.

**Methods:**

A database of 386 patients, enrolled in a randomized clinical trial on the effect of adjuvant chemotherapy (CMF), was analysed. The probabilities of recurrence and death were estimated by the Fine and Gray’s model and by the Cox model. Time dependent covariate and interaction effects were investigated by additive models. Absolute risk reductions (ARR) related to adjuvant treatment or to tumour size [diameter ≤ 2 cm (T1) or >2 cm (T2/T3)] were estimated.

**Results:**

CMF-related reduction in recurrence emerges early, reaches a maximum level at 3 years and persists at a constant level thereafter. Tumour-size-related recurrence reduction, after a maximum at 3 years, displays a progressive regular reduction approaching zero. Patients with any tumour size, when given CMF, exhibit mortality reduction that displays an early regular increase and continues to a persistent plateau. In contrast, tumour-size-related mortality reduction reaches a maximum at 5–7 years and then regularly drops to very low values for patients of both trial arms.

**Conclusions:**

Findings reveal that there is a different time-dependent benefit from chemotherapy and from smaller tumour size at diagnosis. The benefit from adjuvant chemotherapy is long-lasting for patients with any tumour size while the early benefit of diagnosing smaller tumours substantially decreases afterwards. Treatment improvements have probably had greater impact on the mortality reduction than mammography screening.

## Background

The use of adjuvant chemotherapy and hormonal therapy for early breast cancer has strongly increased over the last three decades, mainly following the worldwide overview analyses of randomised clinical trials assessing the effects of adjuvant therapy, showing significant benefits in disease-free and overall survival [1, 2]. Survival improvements associated with the use of adjuvant therapies were estimated to be reductions in the annual odds of death ranging from 8% to 28%, depending on the type and duration of therapy, the age of the patients, and the characteristics of the tumour [1, 2].

Breast cancer mortality was relatively stable until the late 1980s and subsequently slowly and steadily declined in Canada, USA and several countries of Europe [3]. This reduction in mortality cannot be attributed to declines in breast cancer incidence since that has been increasing during the past 30 years in North America and Europe [3, 4]. Thus, reductions in breast cancer mortality, which has been improving from the early 1990s, have been attributed largely to the prolongation of life among women with the disease [4, 5]. Although reasons for the reduction in mortality have been the object of much debate, improvements in early detection and/or improvements of treatments are currently accepted as leading explanations [6, 7].

Analyses of factors responsible for the observed breast cancer mortality decline are difficult to conduct on a population basis because tumour registries often have little information on characteristics of women, their tumour and treatments [5]. Without such information, it is impossible to disentangle the effects of early detection from that of treatments, while simultaneously taking into account secular trends in characteristics of women or their disease. The question has also been addressed by mathematical models [7, 8] for which, however, slight variations in modelling assumptions can result in marked changes in estimated effects, even when common sources of data are adopted [7]. A qualitative agreement between models confirms that both screening and therapy contributed to the decline in mortality although the separate quantitative contributions remain elusive.

There has been extensive analysis of screening mammography effectiveness (e.g. methodological difficulties in trial project and analysis, selective detection of certain types of tumours and breast cancer over-diagnosis) [9]. Nevertheless, screening results in an established effect, i.e. the increased frequency of smaller tumour size at the time of diagnosis that has been observed in every trial that has been conducted. Furthermore, given that a substantial number of breast cancer patients are given adjuvant treatments, there is a crucial question involving the relative improvements in patient prognosis resulting from the reduction in tumour size and the administration of systemic treatment when jointly accomplished. In this report, we indirectly approached this question in the quite specific “experimental” setting of patients with early breast cancer with axillary node involvement, by assessing adjuvant treatment effectiveness according to tumour size at diagnosis in a randomized clinical trial comparing adjuvant chemotherapy to no post-surgical systemic treatment. We considered that, although limited to chemotherapy only and to a particular clinical setting that is much simpler than the population level, the study could provide some useful information about this complex issue.

## Methods

The classical randomized clinical trial carried out at the Istituto Nazionale Tumori in Milan, Italy, to ascertain the effectiveness of adjuvant Cyclophosphamide, Methotrexate and Fluorouracil (CMF) in comparison to no post-mastectomy systemic therapy [10, 11] was analysed. The protocol design was approved by both the Scientific Committee and the Ethic Committee of the Istituto Nazionale Tumori and informed consent was obtained from all patients. It should be emphasized that no mammographic screening was underway at that time.

Patients were stratified according to age, the number of axillary nodes involved, and the type of radical mastectomy (conventional or extended) and were then randomly assigned to receive either CMF for 12 cycles or no further treatment. Neither postoperative irradiation nor adjuvant endocrine therapy was administered.

A total of 386 patients were enrolled: 179 surgery alone and 207 surgery plus adjuvant chemotherapy. CMF treatment consisted of the cyclic administration of Cyclophosphamide (100 mg per square meter of body-surface area orally from day 1 to 14), Methotrexate (40 mg per square meter intravenously on days 1 and 8), and Fluorouracil (600 mg per square meter intravenously on days 1 and 8). Each cycle was followed by a two-week rest period (day 15 to 28). Chemotherapy was started two to four weeks after mastectomy.

Before surgery, all patients underwent a complete physical examination, x-ray study of the chest and skeleton (skull, spine, pelvis, and upper third of femurs), bilateral mammography, differential blood count with platelet count, and biochemical tests. In the absence of symptoms, physical examination was performed every 3 weeks during the first year, every 6 months for the next 4 years, and every 12 months for the following 10 years. Biochemical tests, chest X-ray, and bone X-ray or bone scanning were performed every six to eight months during the first five years and yearly thereafter. Mammography was planned once a year. After the 15th year of follow-up, the patients were examined every 12 to 18 months. In patients with suspicious or controversial radiologic findings, examinations were performed more often. Liver ultrasound scan was performed only if there were suspicious clinical or biochemical findings. Detailed description of patient characteristics is reported elsewhere [10, 11].

Event timing was calculated from the date of surgery. Recurrence was considered to have occurred with the first documented evidence of new manifestations of disease in loco-regional areas (including homolateral supraclavicular adenopathy), distant sites or any combination of these sites. Contralateral breast tumours, second primary cancers and deaths during complete clinical remission were not considered recurrence. Recurrences were analysed considering deaths during complete clinical remission as competing risk. Death from all causes was used as the end-point for overall survival.

Cox model was used to estimate the probability of death according to tumour size at diagnosis and adjuvant treatment. The presence of time by covariates interaction (time-dependent covariate effects either linear, log or spline transform) and between covariates interaction (also time-dependent) were tested using likelihood ratio or Wald test. Absolute risk reduction (ARR) due to adjuvant treatment was estimated for the different tumour sizes. ARR for small tumour size compared to large tumour size was also estimated for patients receiving and not receiving adjuvant treatment. Non-parametric bootstrap was used for confidence interval of estimated survival curves. The same calculations were performed for recurrences using the Fine and Gray’s model.

The additive model was also used to investigate time dependent covariate and interaction effects, both for overall survival, using the Aalen model [12], and recurrences considering competing risks, using the Scheike model [13]. The additive modelling approach allows investigating each covariate effect non-parametrically, i.e. considering each effect non-constant through time. Covariate effects are not multiplicative on the hazard scale, as for Cox model, but additive, and usually results are displayed considering the cumulative hazard function.

## Results

Main patient characteristics are reported in Table 1. In particular, the frequency of tumours with diameter of 2 cm or less (T1) and larger tumours (T2/T3) are well balanced in the two arms. The median (and minimum) follow-up time was 15.8 years.

**Table 1:** **Main patient characteristics**

Characteristic	Controls	CMF
**Number of patients**	179	207
**Tumour size**		
T1	95	103
T2/T3	84	104
**Axillary positive nodes**		
1–3	126	140
> 3	53	67
**Menopausal status**		
Pre-menopause	87	104
Post-menopause	92	103

T1: tumour size ≤ 2 cm; T2/T3: tumour size > 2 cm.

According to Fine & Gray’s model for the analysis of recurrences, evidence for a time-dependent effect (natural cubic spline function of time) for both tumour size and adjuvant therapy was found (p-value for time by tumour size interaction: 0.007; p-value for time by adjuvant therapy interaction: 0.037). However, no evidence for interaction (even time-dependent) between tumour size and CMF treatment was found (p = 0.64). Similar results were found according to the Scheike additive model (tumour size, p = 0.001; adjuvant therapy, p = 0.007; interaction, p = 0.33).

**Figure 1:**
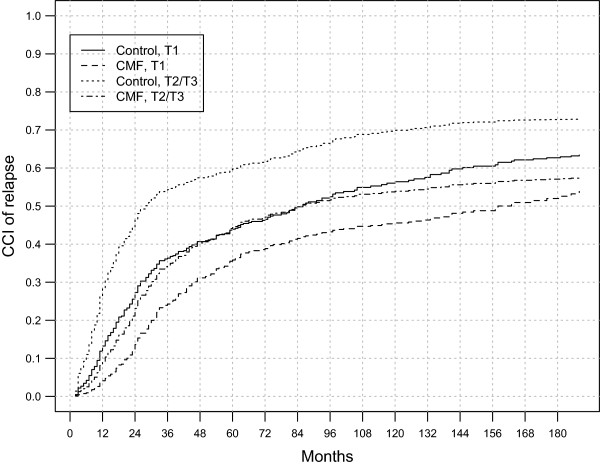
**Probabilities of recurrence estimated using the Fine and Gray’s model with time dependent covariate effects for patients with different tumour size and randomized in the two trial arms.**

**Figure 2:**
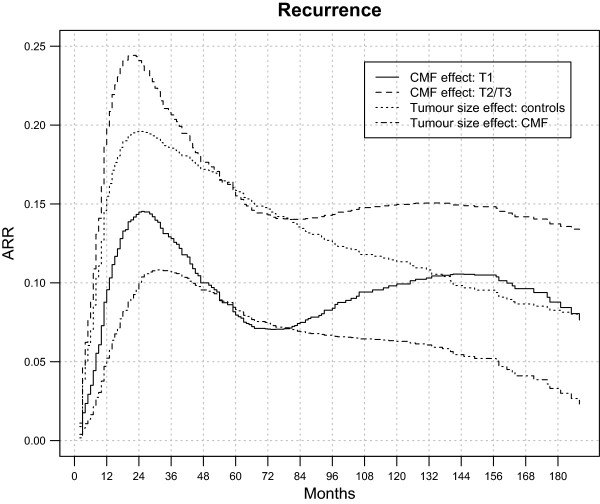
**Adjuvant-CMF-related and tumour-size-related absolute recurrence risk reduction (ARR) for 386 early breast cancer patients undergoing mastectomy only or mastectomy plus adjuvant CMF.** Adjuvant-CMF-related ARR = Recurrence risk for controls - Recurrence risk for CMF treated patients. Tumour-size-related ARR = Recurrence risk for T1 – recurrence risk for T2/T3.

Figure 1 displays the probability of recurrence through follow-up time, sorted by tumour size, of patients from the two trial arms, estimated through the Fine and Gray’s model. Table 2 reports the same probabilities with their 95% confidence intervals at three different follow-up times. To better distinguish the dynamics of the clinical impact due to tumour size and adjuvant treatment, ARRs through the analysed 15 years of follow-up are reported in Figure 2. The analysis of all these quantitative data supports the following statements:

**Table 2:** **Probability of recurrence of patients from the two trial arms, sorted by tumour size, at different follow-up times (95**% **confidence intervals in parenthesis)**

	Years of follow-up		
	5	10	15
Controls, T1	0.46 (0.38-0.54)	0.61 (0.52-0.69)	0.67 (0.59-0.75)
Controls, T2/T3	0.62 (0.53-0.70)	0.72 (0.64-0.80)	0.76 (0.67-0.83)
CMF, T1	0.38 (0.30-0.46)	0.51 (0.42-0.59)	0.59 (0.50-0.67)
CMF, T2/T3	0.46 (0.38-0.55)	0.57 (0.49-0.65)	0.62 (0.54-0.70)

- ➢ Administration of adjuvant CMF reduces the cumulative recurrence rate in both patients with T1 and T2/T3 tumours. The reduction is time-dependent on the relative hazard scale, and ARR shows an early increase to a maximum value at the third year and then decreases to a plateau, which is reached at about the fifth year and persists afterwards up to the end of the observed follow-up. The long-lasting recurrence reduction due to CMF is about 14-16% for patients with T2/T3 tumours and 8-10% for patients with T1 tumours. As already indicated, CMF-related reductions in the two patient subsets are not significantly different.
- ➢ T1 instead of T2/T3 tumour size at diagnosis has impact on the recurrence rate in both patients treated with CMF and controls. In both cases the recurrence reduction on the relative hazard rate scale is time-dependent. ARR maximum value is reached nearly at the third year and displays a progressive and fairly regular reduction afterwards, at least until the end of the analysed time span, when it is apparently approaching zero.

According to the Cox model for the analysis of mortality hazard, evidence for a time-dependent effect (linear) for tumour dimension was found (p-value for time by tumour size interaction: 0.007; p-value for time by adjuvant therapy interaction: 0.62). No evidence for interaction (even time-dependent) between tumour size and CMF treatment was ascertained (p = 0.68). Similar results were obtained according to Aalen additive model as well (tumour size, p = 0.04; adjuvant therapy, p = 0.48; interaction, p = 0.67).

**Figure 3:**
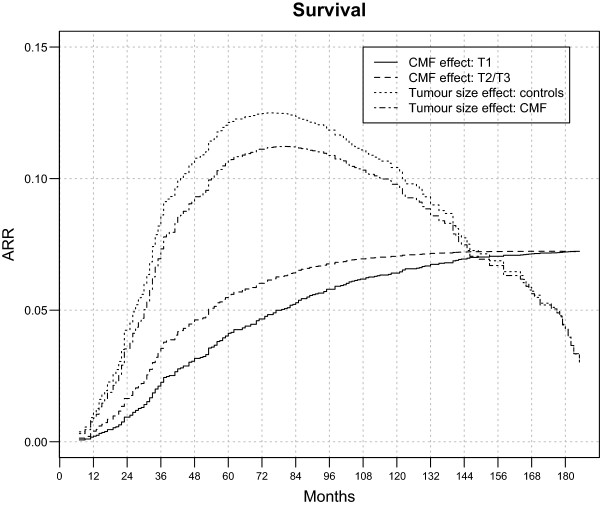
**Adjuvant-CMF-related and tumour-size-related absolute mortality risk reduction (ARR) for 386 early breast cancer patients undergoing mastectomy only (179 patients) or mastectomy plus adjuvant CMF (207 patients).** Adjuvant-CMF-related ARR = Mortality risk for controls - Mortality risk for CMF treated patients. Tumour-size-related ARR = Mortality risk for T1 – Mortality risk for T2/T3.

Table 3 displays the fraction, sorted by tumour size, of patients from the two trial arms who died within different follow-up times, estimated through the Cox model. To indicate the dynamics of the impact on mortality due to tumour size and adjuvant treatment, ARRs through the analysed 15 years of follow-up are reported in Figure 3. The analysis of all these quantitative data supports the following statements.

**Table 3:** **Probability of death of patients from the two trial arms, sorted by tumour size, at different follow-up times (95**% **confidence intervals in parenthesis)**

	Years of follow-up		
	5	10	15
Controls, T1	0.26 (0.20-0.33)	0.47 (0.39-0.55)	0.64 (0.56-0.72)
Controls, T2/T3	0.38 (0.31-0.46)	0.58 (0.49-0.66)	0.68 (0.60-0.76)
CMF, T1	0.22 (0.16-0.28)	0.41 (0.33-0.48)	0.56 (0.48-0.64)
CMF, T2/T3	0.33 (0.26-0.40)	0.51 (0.44-0.59)	0.61 (0.53-0.68)

- ➢ Administration of adjuvant CMF reduces the cumulative mortality in patients with both T1 and T2/T3 tumours. The reduction displays an early regular increase to a plateau persisting up to the end of the observed follow-up. The long-lasting mortality reduction due to CMF is about 7-8%, independent on the tumour size.
- ➢ T1 instead of T2/T3 tumour size at diagnosis has a nearly similar effect on the mortality of patients of both trial arms. The mortality reduction reaches its maximum (12-13%) at 5–7 years. Although this maximum reduction is notably larger than the corresponding above reported reduction due to CMF administration, it steadily decreases afterwards to very low values at the end of the analysed follow-up span.

## Discussion

As previously reported [10, 11, 14], adjuvant CMF, when administered to node positive patients, effectively increases the rate of living patients and patients free from disease at all follow-up times up to 30 years. Here we add the detail that this positive effect on the prognosis displays time-dependence. The recurrence reduction emerges early during the follow-up, reaches its maximum level at about 3 years, declines slightly during the following 3–4 years and persists at a constant level thereafter (Figure 2). This pattern is in agreement with the previous finding that the effect of adjuvant chemotherapy on recurrence dynamics is confined to the first four years following primary tumour removal [15] and to the concept that the benefit it provides to a number of patients is long-lasting (putative cures). The mortality reduction has an early regular increase to a plateau persisting up to the end of the observed follow-up, once again in agreement with the concept of putative CMF related cures. Although reductions in recurrence and mortality are larger for patients diagnosed with tumour diameter more than 2 cm than for patients with smaller tumours, the differences observed in the present database are not statistically significant.

Smaller tumour size at diagnosis elicits better recurrence-free survival for both untreated patients and patients receiving adjuvant CMF (Figure 2). This positive effect is time-dependent and displays an early rapid increase until the third year, when the gain is utmost, but then drops regularly afterwards, without apparently any trend towards a further plateau. The corresponding mortality associated to smaller tumours at diagnosis is somewhat similar for both arms of the randomized trial and displays a trend to vanishing with increasing follow-up time, compared to persistence of CMF related improvement (Figure 3). That a) the same behaviour is detectable in both subsets of treated and control patients and b) similar time-dependent behaviour was previously reported for another breast cancer series [16] contributes evidence to the view of a vanishing effect. Although findings may be related to the fact that tumour size influences only the early recurrence risk (one to four years) [17], other explanations may not be excluded.

The results of this study show a different time-dependent impact on the prognosis from chemotherapy and from tumour size. Although both factors display similar early time dependence, the benefit from adjuvant chemotherapy appears long-lasting for patients with any tumour size, while the early benefit of a smaller tumour size appears to drop to lower values afterwards for patients of both trial arms.

The results of our study may be used for cautious reasoning about mammography screening. The evaluation of the role of mammography screening in the breast cancer clinical approach is quite complex and involves not only biological, clinical and statistical issues but even financial, political and professional conflicts of interest that poison the debate. Despite eight randomised trials including more than 600,000 women, there are still diverging and acrimonious views about the quantification of the benefits of screening and screening recommendations [9]. “Screening saves lives” is strongly claimed in one side [18], while “Which country will be first to stop mammography screening?” is the question from the opposite side [19]. Although the mortality reduction is currently believed (even from screening supporters) to be about half of the initially claimed 30-35% [20–22], the contribution of mammography screening to the general mortality decline has often been considered substantial [23, 7]. The present investigation may provide a few elements that could be useful in the ongoing debate, although indirectly, inferring the effect of diagnosing smaller tumours from the effect of tumour size.

The observed time-dependence of chemotherapy and of tumour size effects may introduce a novel question when comparing benefits from chemotherapy and from diagnosing smaller tumours. Indeed, the long-lasting effect of chemotherapy and the progressively dropping and possibly vanishing benefit of detecting smaller tumours at diagnosis that we observed in this clinical trial should be taken into account when comparing the effects of the two factors at population levels.

In this study, we evaluated quantitative improvements under the assumption that all tumours have sizes of 2 cm or less instead of more than 2 cm. If one would like to translate our evaluations to tumour size reductions due to mammography screening, this assumption might be too restrictive. Indeed, only a fraction of tumours detected by screening procedures may be down-graded from T2/T3 category to T1 category. From the frequency distribution of sizes of breast cancers detected in Sweden and Canada [24] and in Edinburg [25] in women invited for mammography and in controls, it can be estimated that the percentage of tumour size down-staging ranges from 10% to 24%. Therefore, the above mentioned expected benefits should be considered accordingly.

The study was carried out in a quite particular setting. Indeed, although randomized clinical trials have demonstrated survival benefits associated with the use of adjuvant treatments, depending on the type and duration of therapy, the age of the patients, and the characteristics of the tumour [1], the extent to which these benefits translate to the population outside the controlled conditions of clinical trials is substantially unknown. Also, it should be emphasized that present findings from node positive patients could not be “per se” translated to women with node negative disease. Moreover, the analysed database regarded women given adjuvant CMF only, without any other adjuvant treatment. Therefore, the study could not ascertain the effect of tumour size on the prognosis of patients either receiving more recent combination treatments (e.g. doxorubicin and/or taxane based and HER2 targeting treatments) or adjuvant hormone treatments or a combination of both. It may be reasonably alleged, therefore, that our results may somewhat underestimate the effect of adjuvant systemic treatments.

The analysed data were collected in an era prior to mammographic screening and CMF chemotherapy is no longer widely used in the adjuvant setting. Furthermore, breast cancers were not classified according to modern biologic subgroups (e.g. triple negative, hormone receptor or Her2 positive). Yet, such facts do not lessen the core of the results. Indeed, a) improvements in adjuvant chemotherapy by new drugs did not change the recurrence dynamics (still unpublished data); b) analysis of recurrence dynamics according to modern biologic subgroups revealed similar pattern, although with different subgroup-related levels of recurrence risk [26, 27].

The results of our analysis should be evaluated keeping in mind a few cautionary considerations. The assumption that mammographic screening reduces the rate of T2/T3 breast cancers, in spite of its current consent, is still lacking actual support. Most of reported data on screening-related late stage reductions are stated as percentages of diagnosed tumours, which are affected by the overdiagnosis phenomenon. Additionally, there are unexpected differences between the results from randomized trials (late-stage reduction) and results from population-based studies (no late-stage reduction) [28, 29], indicative that the question needs further research. A second point at present poorly clarified regards the biology of breast cancer. Indeed, the course of clinically diagnosed tumours might be somehow dissimilar from the course of tumours with the same size detected by screening [30]. This occurrence would introduce, therefore, an additional prognostic factor.

## Conclusion

In conclusion, the results of our hypothesis generating study, which evaluates tumour-size-related prognosis changes in a particular setting, suggest that the effect of tumour size on the mortality reduction may be limited to the early post-surgery time in comparison with the corresponding effect of adjuvant therapies, which seems to play a more prominent and long-lasting role. This view is consonant recent reports [31, 23] showing that in Denmark and Norway (two “model countries” in respect to mammography screening) mortality reductions have been detected in both screened and non-screened areas since early 90’s (when adjuvant therapy was routinely introduced) and that the estimated mortality declines by area were similar [31] or only moderately different with a minor fraction attributable to screening [23]. In the absence of other opposing indications, we suggest that the mortality reduction for breast cancer patients observed during the last decades might be mainly attributed to treatment improvements, even if mammography screening may have been contributory to some extent.
